# Supplementary material for: Cholesterol and Egg Intakes, and Risk of Hypertension in a Large Prospective Cohort of French Women
Source: Nutrients. 2020 May 8;12(5):1350. doi: 10.3390/nu12051350 (PMC7285051; doi:10.3390/nu12051350)
Supplement: Supplementary file 1 [file nutrients-12-01350-s001.pdf]

Supplementary figure 1

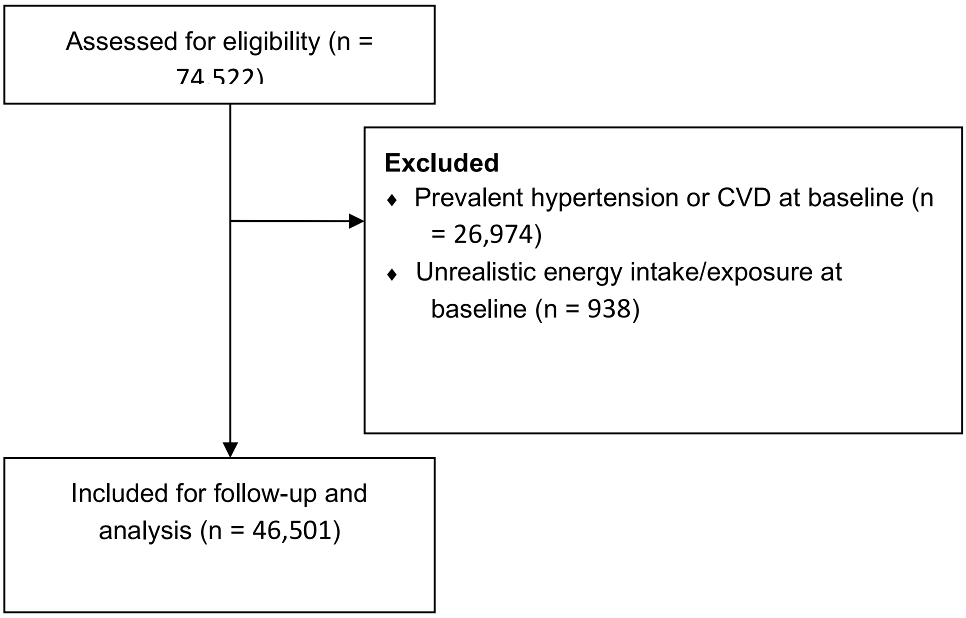

Supplementary table 1

Variables (mean, SD)	Included	Excluded
Eggs / week	2.4 (2.3)	2.4 (2.5)
Cholesterol (mg / day)	369 (141)	372 (163.5)
Age (years)	50.1 (6.3)	53.1 (7.0)
BMI (kg / m^2)	22.3 (2.8)	23.7 (3.7)
Total physical activity (METS / week)	55.3 (29.8)	55.9 (31.1)
Diabetes (%)	0.5	1.6
Dyslipidaemia (%)	5.2	10.5
Family history CVD (%)	33.7	36.1
Prior cancer (%)	6.3	7.4
High school education (%)	54.8	61.0

University education (%)	38.8	30.5
Smoking (Never/X/current) (%)	52.0/33.8/14.3	56.5/31.6/11.9
**Dietary variables (median, SD)**		
Total energy (Kcal)	2077 (534)	2078 (625)
Salt (mg / day)	2732 (898)	2757 (1005)
Potassium (mg / day)	3723 (1030)	3778 (1136)
Meat (g / day)	78 (43)	79 (45)
Processed meat (sausage, ham, pate) (g / day)	16 (18)	17 (20)
Fish (g / day)	25 (22)	25 (23)
Shellfish / canned fish (g / day)	6 (11)	4 (13)

Vegetables (g / day)	143 (102)	143 (106)
Fruit (g / day)	228 (169)	235 (176)
Dietary fibre (g / day)	24 (8)	24 (8)
Alcohol (g / day)	7 (14)	7 (15)
Total fats (g / day)	86 (27)	86 (31)
Prudent diet score [-1, 1]	-0.13 (0.97)	-0.10 (1.0)
